# Antipsychotic use in a resource-limited setting: Findings in an Eastern Cape psychiatric hospital

**DOI:** 10.4102/sajpsychiatry.v23i0.1093

**Published:** 2017-07-18

**Authors:** Ingrid Eloff, Willem Esterhuysen, Kavendren Odayar

**Affiliations:** 1 Faculty of Health Science, Walter Sisulu University, South Africa

## Abstract

**Background:**

Second-generation antipsychotics (SGAs) are commonly prescribed despite the fact that large, naturalistic studies have failed to show superior efficacy and tolerability when compared with first-generation antipsychotics (FGAs). In addition to this, the availability of SGAs in the South African public health sector is limited because of higher acquisition costs. Therefore, judicious use of FGAs, which are affordable and more widely available, should be considered.

**Aims:**

This study aimed to (1) determine how frequently patients are switched from an FGA to an SGA in an acute psychiatric hospital in the Eastern Cape, (2) determine reasons for switching and (3) compare the profiles of the switch group to the non-switch group.

**Method:**

The study is a cross-sectional survey conducted as a retrospective chart review at a psychiatric hospital in the Eastern Cape over a study period of 2 months. The demographics, diagnostic data, antipsychotic drug used and whether a switch from an FGA to an SGA took place were recorded using a data collection document. The sample included 169 patients.

**Results:**

Of the 169 patients, 125 (74%) were initiated on an FGA and 44 (26%) on an SGA on admission. Of the 125 patients who were initiated on an FGA, 43 (34%) were switched to an SGA during the course of the admission. Therefore, 87 (51%) participants were discharged on an SGA. The main reasons for switching were the emergence of extrapyramidal side-effects (EPSE) (63%) followed by lack of efficacy (19%). The only statistically significant difference between the switch and non-switch groups was that the switch group was on average younger than the non-switch group.

**Conclusion:**

SGAs, with the exception of clozapine, have not been proven to be superior to FGAs. Although FGAs are more prone to cause EPSE, SGAs carry significant risks of their own. FGAs are also more freely available and cost effective in South-Africa. Despite these facts the prescribing of and switching to SGAs remain prevalent in our setting with a switch rate of 34% and more than half of our patients being discharged on SGAs.

## Introduction

Antipsychotic medications are used to manage the symptoms of several psychiatric disorders.^1^ These agents are commonly divided into two classes: first-generation antipsychotics (FGAs), also referred to as ‘typical antipsychotics’, and second-generation antipsychotics (SGAs), also known as ‘atypical antipsychotics’.^2^ The former show a high affinity for dopamine 2 (D2) receptors, while the latter are dopamine-serotonin antagonists with a high affinity for serotonin 2A (5HT-2A) receptors.^2^

The introduction of FGAs changed the way that patients with psychotic disorders were managed.^1^ Following the introduction of the early antipsychotics, clinicians began noticing signs of parkinsonism in patients treated with these drugs. This led to a search for effective drugs without the risk of extrapyramidal side-effects (EPSE).^3^

Second-generation antipsychotics were initially thought to be superior to FGAs because of a lower risk of drug-induced movement disorders as well as apparent superior efficacy.^4^ However, the safety advantages of SGAs have been called into question because of their tendency to cause weight gain as well as altering glucose and lipid metabolism.^5^ Further, long-term comparative effectiveness trials such as CATIE (Clinical Antipsychotic Trials of Effectiveness) and CUtLASS (Cost Utility of the Latest Antipsychotic drugs in Schizophrenia Study) and meta-analyses have challenged the idea that these drugs are more efficacious than the FGAs.^4^ While some studies have reported modest symptom gains, conclusive data with regards to improvement on objective measurement scales of functioning are lacking.^6^

The side effects of both FGAs and SGAs can have a detrimental impact on quality of life, treatment adherence, relapse rates and stigma.^7^ Although both FGAs and SGAs are heterogeneous classes, FGAs are more likely to cause EPSE while SGAs have a greater tendency to lead to weight gain and metabolic side effects.^8^

It has been noted in some studies that FGAs may be cost saving when compared with SGAs and that, in the US, most on-patent SGAs cost ten to one hundred times more than older drugs.^6^ This may be an important consideration in our setting because of the fact that for many low- and middle-income countries (LAMICs) such as South Africa, access to the SGAs is limited given their relative higher cost.^9^

Furthermore, given the enormity of the non-adherence problem in our study setting, conventional long-acting depot antipsychotics may be an effective as well as cost-effective way of managing patients with psychotic disorders.

Although antipsychotic switching is common in clinical practice, there is paucity of data on the clinical value of various switching approaches.^10^ In 2009, the National Institute of Mental Health-funded Schizophrenia Patient Outcomes Research Team (PORT) published updated recommendations for the treatment of schizophrenia. With regards to acute antipsychotic treatment, the studies reviewed suggested that there were no data to support a switch to an SGA in patients whose symptoms are controlled with minimal side effects on an FGA.^11^

## Aims

This study aimed to determine how frequently patients’ medication was switched from an FGA to an SGA in an acute psychiatric hospital in the Eastern Cape. It also aimed to ascertain the reasons for switching as well as to compare the demographic and clinical profiles of the switch group with that of the non-switch group.

## Methodology

### Study design and setting

This study was a retrospective chart review with a cross-sectional design. It was conducted at a designated psychiatric hospital in the Eastern Cape. Data were extracted from the files of patients who were admitted to the hospital from 01 November 2012 to 31 December 2012 and were statistically analysed.

### Study population and sampling

#### Study population

The study population consisted of patients who were admitted to a designated psychiatric hospital in the Eastern Cape in terms of the *Mental Health Care Act* no.17 of 2002. Patients admitted to the hospital are predominantly those with a serious mental illness (SMI) which includes, but is not limited to, schizophrenia and other psychotic disorders, bipolar disorder as well as substance-related psychotic and mood disorders.

#### Inclusion criteria

All patients who were admitted to the psychiatric hospital during the study period and were treated with antipsychotic medication were eligible for inclusion in the study.

#### Sample size

The files of all patients who were admitted to the hospital from 01 November 2012 to 31 December 2012 were collected and examined. This gave a sample size of 169 patients.

### Data collection

All files contained the doctors’ admission notes, follow-up clinical notes and patient prescription charts. It was possible to obtain information on the patient’s diagnosis, reason for admission, duration of admission, medication that was initiated and whether it was changed to another medication. If stated, the reasons given for changing a patient’s medication were recorded.

A data collection sheet was used to document information from each file. The files were reviewed by the primary investigator, and the data collection sheets were completed at the same time. No files were removed from the hospital premises for data collection. Following collection, data were electronically stored in a secure, password-protected electronic database system. A sequential number was allocated to each participant to distinguish questionnaires and to ensure anonymity and preserve confidentiality.

The data collection document was designed to collect the following broad categories of data: demographic data, diagnostic data and medication of current admission:

- **Demographic data:** This category included the age, sex, race and number of admissions.
- **Diagnostic data:** This category included psychiatric diagnosis (DSM-IV-TR as was in use during the study period), medical comorbidity and substance use.
- **Current admission medication:** This category captured whether an FGA was initiated for treatment purposes at any point during the chosen study period (current admission) and if so whether the FGA was switched to an SGA. Data were also captured regarding whether a reason for switching was stated and what the reason was.

### Analysis of data

Data from the data collection sheet were captured electronically by the researcher in Microsoft Excel. Further analysis was carried out by using SAS Version 9.2. Descriptive statistics, namely, frequencies and percentages, were calculated for categorical data. Furthermore, the effects of the patient’s demographic and diagnostic information on the prescribing habits were investigated. Analytical statistics, namely, the chi-square test (or Fisher’s exact test) for nominal data and the Kruskal-Wallis test for ordinal data, was used. A significance level of 0.05 was used.

## Ethical consideration

Ethics approval was obtained from the Biosafety and Ethics Committee of Walter Sisulu University. Written permission to conduct research at the hospital was obtained from the chief executive officer of the hospital.

## Results

### Sample size

A total of 169 participants met the inclusion criteria.

### Use of antipsychotics

**FIGURE 1:**
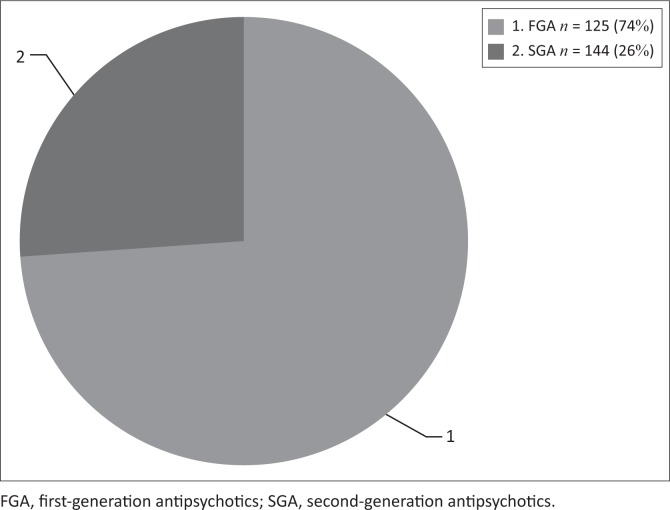
Antipsychotic initiated on admission.

Of the 169 (*n* = 169) participants, all (100%) were started on antipsychotic medication on the day of admission. Of the 169 patients in the study, 125 (74%) were initiated on an FGA and haloperidol was chosen in all 125 of these cases. The remainder, 44 (26%) were initiated on an SGA (Figure 1).

### Frequency of switching from first-generation antipsychotic to second-generation antipsychotic

**FIGURE 2:**
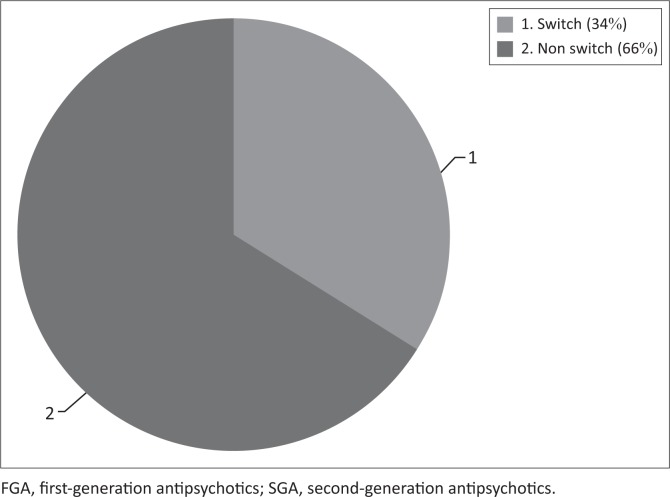
Percentage of patients who remained on FGA and percentage of patients who were switched to SGA.

**FIGURE 3:**
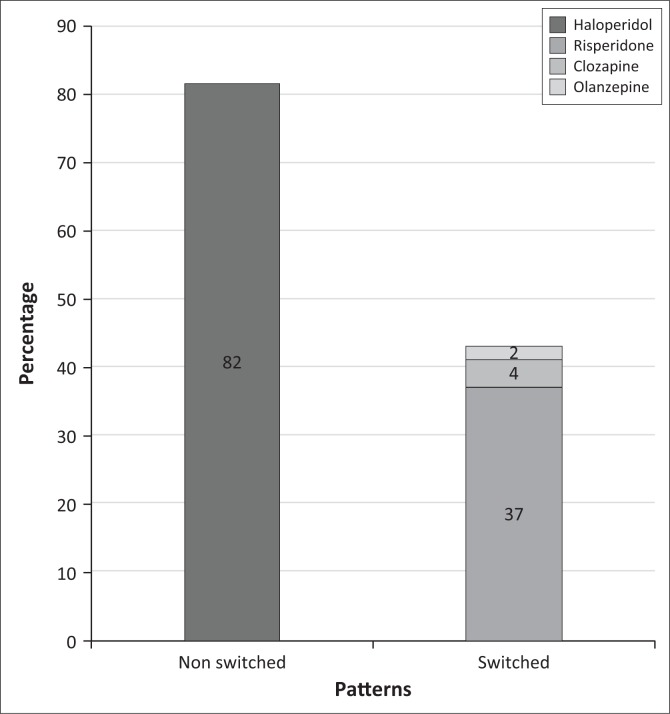
Patterns of antipsychotic use.

In the group of patients who were initiated on an FGA (*n* = 125), 43 patients (34%) were switched to an SGA while 82 (66%) remained on an FGA. Of the switches, 37 (86%) were switched to risperidone, 4 (9%) to clozapine and 2 (5%) to olanzapine. In this group of patients who were switched to an SGA, 22 (55%) were switched in less than 2 weeks after the initiation of the FGA (Figures 2 and 3).

### Reasons for switching from first-generation antipsychotic to second-generation antipsychotic

The reason for switching was clearly stated for 27 (63%) participants in the switch group. Therefore, for 16 (37%) of the patients in the switch group, reasons for switching were not explicitly stated. In the group of patients where no reason for switching was explicitly stated, the researcher attempted to extrapolate possible reasons by examining clinical notes made by the treating physician and was able to do this for 14 patients. The researcher was unable to identify any reasons for switching from the remaining two files.

In the group where the reason for the switch was stated (*n* = 27), the main reason given by clinicians for switching was EPSE (*n* = 22; 81%), followed by lack of efficacy (*n* = 4; 15%). One patient (4%) was switched because of patient preference.

In the group where the researcher deduced reasons for switching from the file (*n* = 14), the reasons were EPSE (*n* = 5; 42%), lack of efficacy (*n* = 4; 33%), previous response to an SGA (*n* = 3; 25%), mood symptoms (*n* = 1; 8%) and catatonia (*n* = 1; 8%).

**FIGURE 4:**
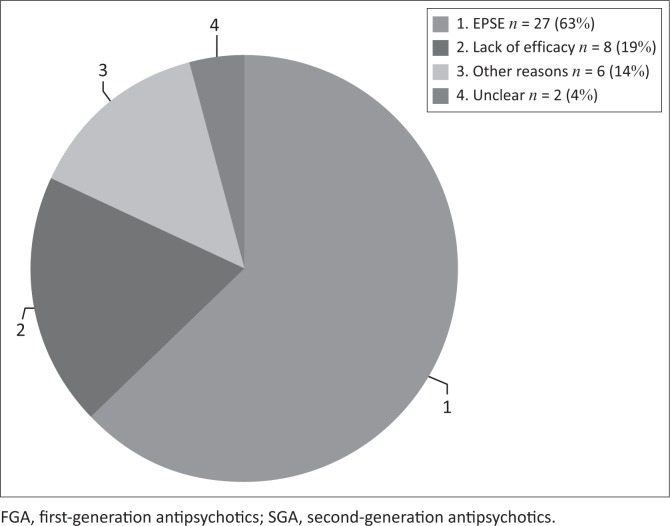
Reasons for switching from FGA to SGA.

Therefore, in the switch group of 43 patients, 27 (63%) were switched because of EPSE, 8 patients (19%) because of lack of efficacy and 6 patients (14%) for other reasons, and for 2 (4%) participants no reason for switch was given (Figure 4).

### Comparison of the switch group with the non-switch group

**FIGURE 5:**
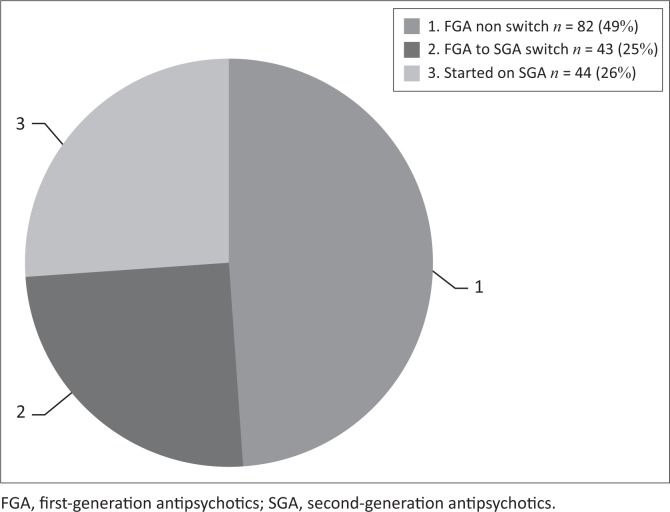
Patterns of antipsychotic use, three distinct groups.

From the study sample (*n* = 169), one could distinguish three distinct groups of patients based on antipsychotics prescribed. The first group consisted of the patients who were initiated on an FGA on admission, remained on that FGA and were eventually also discharged on it. The number of patients in the ‘FGA only’ group was 82 (49%). The second group included the patients who were switched from an FGA to an SGA (43 patients; 25%). The third group (44 patients; 26%) was the patients who were initiated on an SGA and remained on an SGA. Within the third group, there were participants who were switched from one SGA to another (Figure 5).

Within the total study sample, there were no participants who were switched from an SGA to an FGA.

In order to compare the FGA only group (non-switch group) and the FGA to SGA group (switch group), the effects of the patient’s demographic and diagnostic information on the selection of an antipsychotic were statistically analysed.

When looking at sex, race or number of admissions there were no statistically significant differences between the non-switch and the switch groups.

However, there was a statistically significant difference in age ( *p* = 0.0314) in that the non-switch group was on average older than the switch group.

**FIGURE 6:**
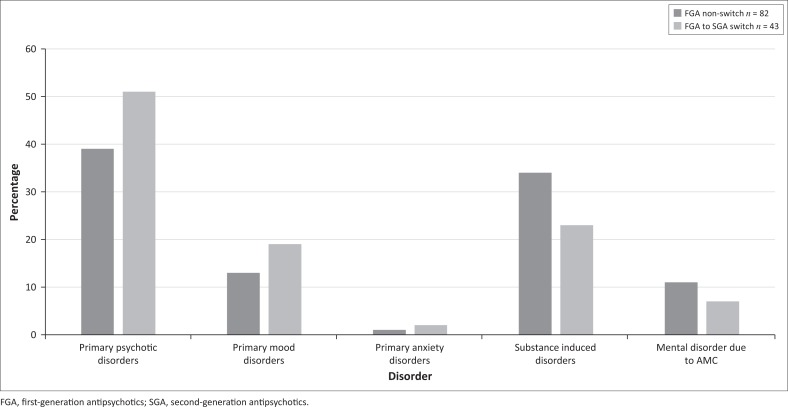
Diagnostic distribution of switch and non-switch groups.

When assessing the diagnostic data of the two groups, there was a similar distribution with no statistically significant differences (Figure 6).

## Discussion

Of the 169 participants included in the study, almost three quarters (*n* = 125, 74%) were initiated on an FGA on the day of admission to the hospital, with haloperidol being the agent of choice for the entire group. Possible factors that may have influenced the high prescription rates of haloperidol in the index study may include low cost and availability of haloperidol in our setting. Based on the January 2016 pricelist of the pharmaceutical distributing depot which supplies the hospital in this study, the cost of 2.5 mg haloperidol for a single patient for 1 month is R3.66. A study published in the *Bulletin of the WHO* in 2008 looking at the treatment of schizophrenia in the developing world found that treatment with an FGA was the most cost effective.^12^

Of those participants that were initiated on an FGA (*n* = 125), approximately one third (*n* = 43; 34%) were switched to an SGA during the course of their admission. The lack of pre-existing data on switch rates from FGAs to SGAs makes comparative estimates virtually impossible. An important principle mentioned in literature is that before going the switch route, prescribers should consider taking steps (such as dose adjustments) to optimise current medication regimens.^13^ In our study, switching from an FGA to an SGA tended to take place early on in the course of treatment. The majority (*n* = 24; 56%) of the patients in the switch group were switched to an SGA within less than 2 weeks on treatment. This finding is noteworthy, as 2 weeks does not constitute an adequate trial of an antipsychotic.^14^ Possible reasons for this may have been the emergence of acute EPSE, which tend to occur within days after initiating an FGA.^15^ These side effects could potentially be managed by reducing the dose of treatment and/or the judicious use of anticholinergic drugs.^14^ This may be a step that could be taken before switching a patient from an FGA to an SGA.

From this study, it is clear that EPSE is an important reason for switching from FGAs to SGAs, with 63% of the switches occurring because of the development of EPSE. Lack of efficacy accounted for only 19% of the switches. This is in keeping with data that have shown that despite heterogeneity within the classes, FGAs (particularly high potency FGAs) have a higher risk of causing EPSE than SGAs.^8^ This finding highlights a number of important matters. Careful assessment and documentation of drug-induced movement disorders using objective rating scales is essential. Early identification of EPSE may allow for appropriate dose adjustments to be made. Although this study did not investigate drug doses some studies have found that low doses of FGAs may be effective and well-tolerated.^16^ This could potentially be an option in our setting.

A large proportion of patients in this study (26%) were initiated on an SGA on admission without being given a trial of an FGA. For 21 (48%) of participants in this SGA-only group it was a first admission. This is an important finding as a recent study conducted in the Western Cape has shown that low dose depot FGA together with assertive monitoring programmes may be a safe and effective intervention for patients with first episode psychosis.^17^ Further studies at this hospital could aim to examine the use of antipsychotic drugs in the first episode of psychosis.

In our total study sample of 169 participants, 43 patients were switched to an SGA and 44 participants were initiated on an SGA from admission. This means that 87 (51%) of the patients in this study were discharged from the hospital on an SGA. Although this finding is in keeping with worldwide antipsychotic prescribing trends,^6^ it may have significant cost implications in our resource-constrained setting.

## Limitations and recommendations

There are limitations to this study including its retrospective design with convenience sampling, which may have resulted in bias. Another factor is the relatively small number of treating clinicians working at the hospital at the time the study was conducted. This may limit the ability to make generalisations about prescribing habits. The dosages of FGAs and SGAs used were not recorded in this study. This would have been valuable information to ascertain at what dosage of an FGA patients were switched to an SGA. The concomitant use of anticholinergic drugs used for the treatment of EPSE was also not recorded on the data collection sheet. Without this information, it is difficult to draw conclusions about whether FGA doses correlate with the emergence of EPSE and whether anticholinergic drugs were used appropriately for the alleviation of EPSE before antipsychotics were switched.

Confounding factors that might have influences on the frequency of switching as well as tolerability of antipsychotics exist, including high prevalence of substance use in the sample and high prevalence of comorbid medical disorders such as HIV.

## Conclusion

Second-generation antipsychotics, with the exception of clozapine, have not been conclusively shown to be superior to FGAs. FGAs are more prone to cause EPSE than SGAs. However, SGAs carry significant risks of their own such as weight gain, diabetes and dyslipidaemia. FGAs are more freely available and cost effective than SGAs in the South-African public health sector. FGA depot preparations are also accessible and affordable in our setting. Given the significant problem of non-adherence, this may be an important factor to consider when choosing antipsychotic medications.

Despite this the prescribing of- and switching to SGAs remain prevalent in our setting. The frequency of switching from an FGA to an SGA in this sample was 34%, and more than half of the participants in the sample were discharged on SGAs. In our resource-constrained setting, this may have significant cost implications.

It would be prudent to further analyse and perhaps adapt our prescribing practices to best suit our patient population and to ensure optimal outcomes for our setting.
